# Ambient noise exposure induces long-term adaptations in adult brainstem neurons

**DOI:** 10.1038/s41598-021-84230-9

**Published:** 2021-03-04

**Authors:** Ida Siveke, Mike H. Myoga, Benedikt Grothe, Felix Felmy

**Affiliations:** 1 Division of Neurobiology, Department Biology II, Ludwig-Maximilians-University Munich, 82152 Planegg-Martinsried, Germany; 2 Institute of Zoology and Neurobiology, Ruhr-University Bochum, Universitätsstrasse 150, 44780 Bochum, Germany; 3 Institute of Zoology, University of Veterinary Medicine Hannover, Foundation, Bünteweg 17, 30599 Hannover, Germany

**Keywords:** Auditory system, Neuronal physiology

## Abstract

To counterbalance long-term environmental changes, neuronal circuits adapt the processing of sensory information. In the auditory system, ongoing background noise drives long-lasting adaptive mechanism in binaural coincidence detector neurons in the superior olive. However, the compensatory cellular mechanisms of the binaural neurons in the medial superior olive (MSO) to long-term background changes are unexplored. Here we investigated the cellular properties of MSO neurons during long-lasting adaptations induced by moderate omnidirectional noise exposure. After noise exposure, the input resistance of MSO neurons of mature Mongolian gerbils was reduced, likely due to an upregulation of hyperpolarisation-activated cation and low voltage-activated potassium currents. Functionally, the long-lasting adaptations increased the action potential current threshold and facilitated high frequency output generation. Noise exposure accelerated the occurrence of spontaneous postsynaptic currents. Together, our data suggest that cellular adaptations in coincidence detector neurons of the MSO to continuous noise exposure likely increase the sensitivity to differences in sound pressure levels.

## Introduction

Neuronal adaptation adjusts the processing of sounds to changes in acoustic environment^1–4^. In the auditory system different mechanism including molecular^5^, structural^6–9^ and functional adaptation^10–15^ contribute to this adjustment. Thus, various cellular mechanisms modulate auditory processing over a large time range covering milliseconds to days.

Here we investigated long-term adaptive changes in neurons of the medial superior olive (MSO). MSO neurons are the major site of coincidence detection of binaural inputs and process binaural temporal information known to be important to localize low frequency sound sources in the azimuth^16,17^. On the cellular level, MSO neurons process inputs by an ultrafast integration mechanism based on fast excitation^18^ and inhibition^19–22^ and voltage-gated postsynaptic conductances^23–28^. Moreover, action potential generation of MSO neurons is facilitated by membrane resonance^29,30^, a phenomenon largely determined by the overall membrane conductance^29^.

Concurrent or masking signals, like ongoing background noise have a substantial effect on binaural processing in the auditory brainstem^14,31,32^. Binaural neurons in the brainstem rapidly adapt to short-term stimulation history within milliseconds^10,11,15^. After long-lasting exposure to moderate omnidirectional noise, that masks sound sources localization cues, long-term reversible adaptations occur for the processing of binaural cues^14^. Thus, noise exposure induces long-term adaptations in the binaural system. The cellular mechanisms behind this adaptation, however, are unknown.

We investigated the biophysical properties of coincidence detector neurons of the MSO that undergo long-term adaptation during omnidirectional noise exposure. Noise exposure leads to a drop in input resistance, an increase in action potential current threshold and a modulation of synaptic inputs. These cellular changes are indicative of an increased sensitivity to differences in sound pressure levels after noise exposure.

## Results

### Effects of noise exposure on biophysical membrane properties

**Figure 1:**
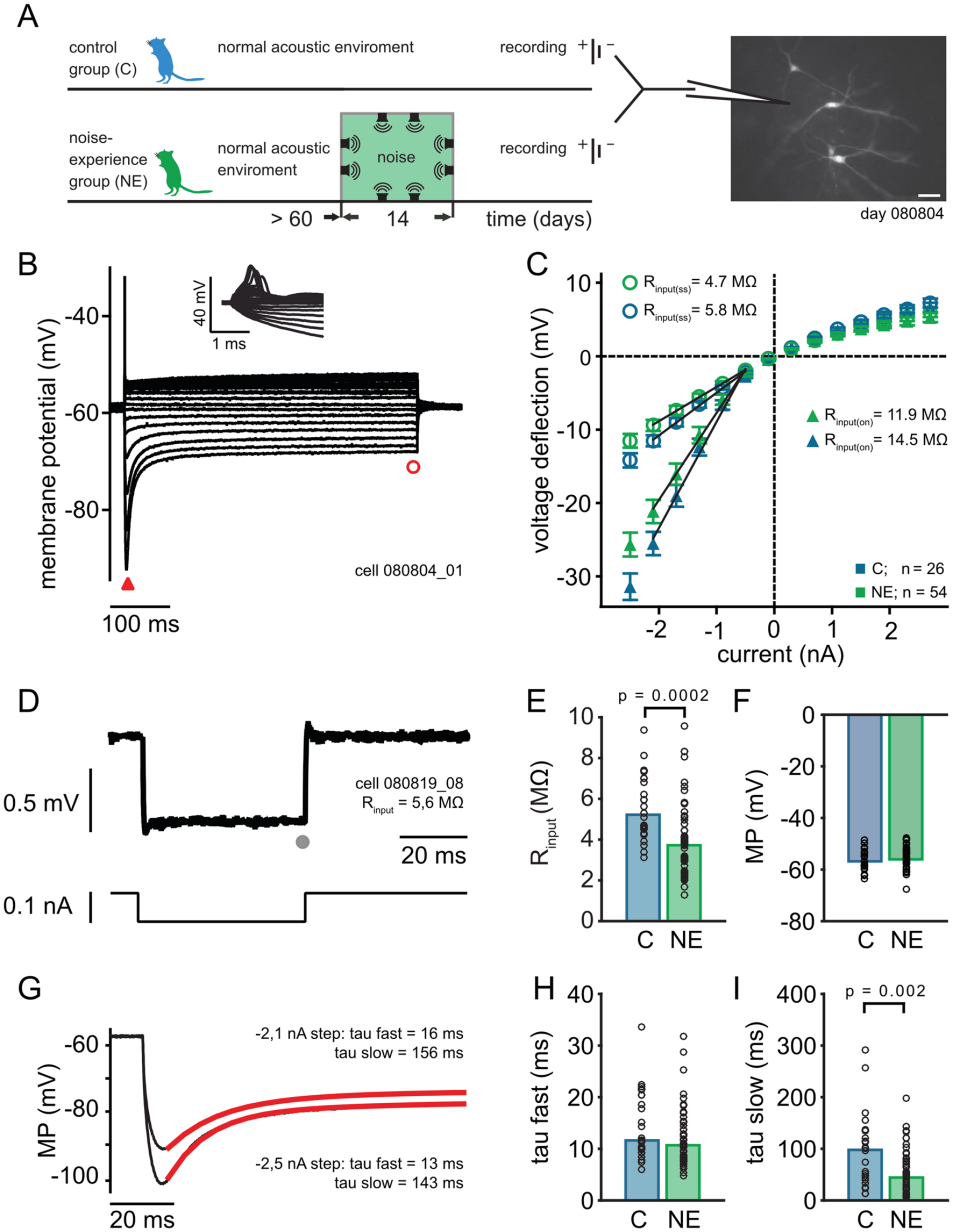
Intrinsic properties of MSO principal neurons from adult animals with and without noise exposure: neurons are getting leaky and faster after noise exposure. (**A**) Schematic drawing of the experimental design and three typical principal MSO neurons filled with Alexa Fluor 568 during recording (right picture). (**B**) Response to different 500 ms current pulses (− 2.5 to 4.3 nA; 0.4 nA steps) using current clamp recordings while blocking synaptic transmission (SR—GABA antagonist, D-AP5-NMDA antagonist, TTX – Na_1_). (**C**), On-current (on; triangle, see (**B**) for example) and the steady-state-current (ss; circle, see (**B**)) V–I plots generated from the average voltage responses of ≥ 3 repetitions. The average R_input_ was estimated from the slope of the V–I plots between − 2.1 and − 0.5 nA current application. (**D**) Response to a small current injection (− 0.1 nA, grey lines) were used to estimate the steady state (grey dot) R_input_ of each cell to the average response (black line; ≥ 20 repetitions). (**E**) The R_input_ of the control neurons (median = 5.2 MΩ) is significant higher (p = 0.0002) as the resistance of neurons of animals exposed to noise (median = 3.7 MΩ). (**F**) The membrane potentials (MP) do not differ between the two groups. (**G**) Example average response (≥ 3 repetitions) to high negative currents (− 2.5 and − 2.1 nA) were used to estimate the membrane potential and the time constants of the depolarizing sag (defined by a double exponential fit, red lines) during negative current injections. (**H**) The fast time constants of the depolarizing sag potential were not changed during noise exposure (c = 11.6 ms; NE = 10.7 ms). (**I**) The slow time constants were significant smaller (p = 0.002) in neurons of noise exposed animals (c = 0.098 s; NE = 0.044 s). Bars represent the median values, significance was assessed using two-sided Wilcoxon rank sum test.

To investigate the long-term adaptations after omnidirectional white noise exposure, we performed whole-cell recordings from MSO neurons in acute brain slices from control (c) and noise experienced (NE) animals (Fig. 1A). We recorded from visually identified neurons in the central band of the MSO. This location was controlled and verified online and by post-hoc visualization (Fig. 1A, right). The biophysical properties were determined with step current-clamp protocols (Fig. 1). As described before^18,26,33,34^, MSO neurons showed a single onset action potential and a large, rapid sag potential (Fig. 1B). The sub-threshold voltage response at the onset and in steady state was analyzed (Fig. 1C). From the voltage-current relationship, the membrane input resistance (R_input_) was estimated by determining the slope between -0.5 and -2.1 nA stimulation current. From the average the estimated R_input_ at hyperpolarized membrane potentials was reduced after the sound exposure at the onset (on) and the steady state (ss) (Fig. 1C; control n = 26; NE n = 54). The decrease of the R_input_ was confirmed (Fig. 1D,E) using the average voltage response to a -100 pA hyperpolarizing current repeated 30 times (control n = 26; NE n = 54; p = 0.0002, two-sided Wilcoxon rank sum test). However, the membrane potential of the neurons was unchanged after noise exposure (Fig. 1F; control n = 26; NE n = 54; p = 0.501, two-sided Wilcoxon rank sum test).

The R_input_ of MSO neurons depends mainly on I_H_ and K_LT_ currents, which are activated at resting potentials^35^. The quantification of the resting membrane potential (control -54.3 ± 0.56 mV; NE − 55.7 ± 0.75 mV) however indicated no significant (p = 0.501) change due to noise exposure. Thus, as both I_H_ and K_LT_ currents contribute to R_input_ at resting potentials, noise exposure might upregulate both currents simultaneously. To indicate long-term changes in I_H_ currents the sag decay evoked by a − 2.4 and − 2.1 nA current injection was fitted by a double-exponential function (Fig. 1G). Noise exposure did not change the fast (Fig. 1H; control 11.6 ms, n = 26; NE 10.7 ms n = 54; p = 0.14, two-sided Wilcoxon rank sum test), but significantly accelerated the slow decay component (Fig. 1I; control 98 ms; NE 44 ms; p = 0.002, two-sided Wilcoxon rank sum test). The acceleration of the decay may indicate that more I_H_ current might be present after moderate, long-lasting omnidirectional noise exposure.

### Effects of noise exposure on action potential generation

**Figure 2:**
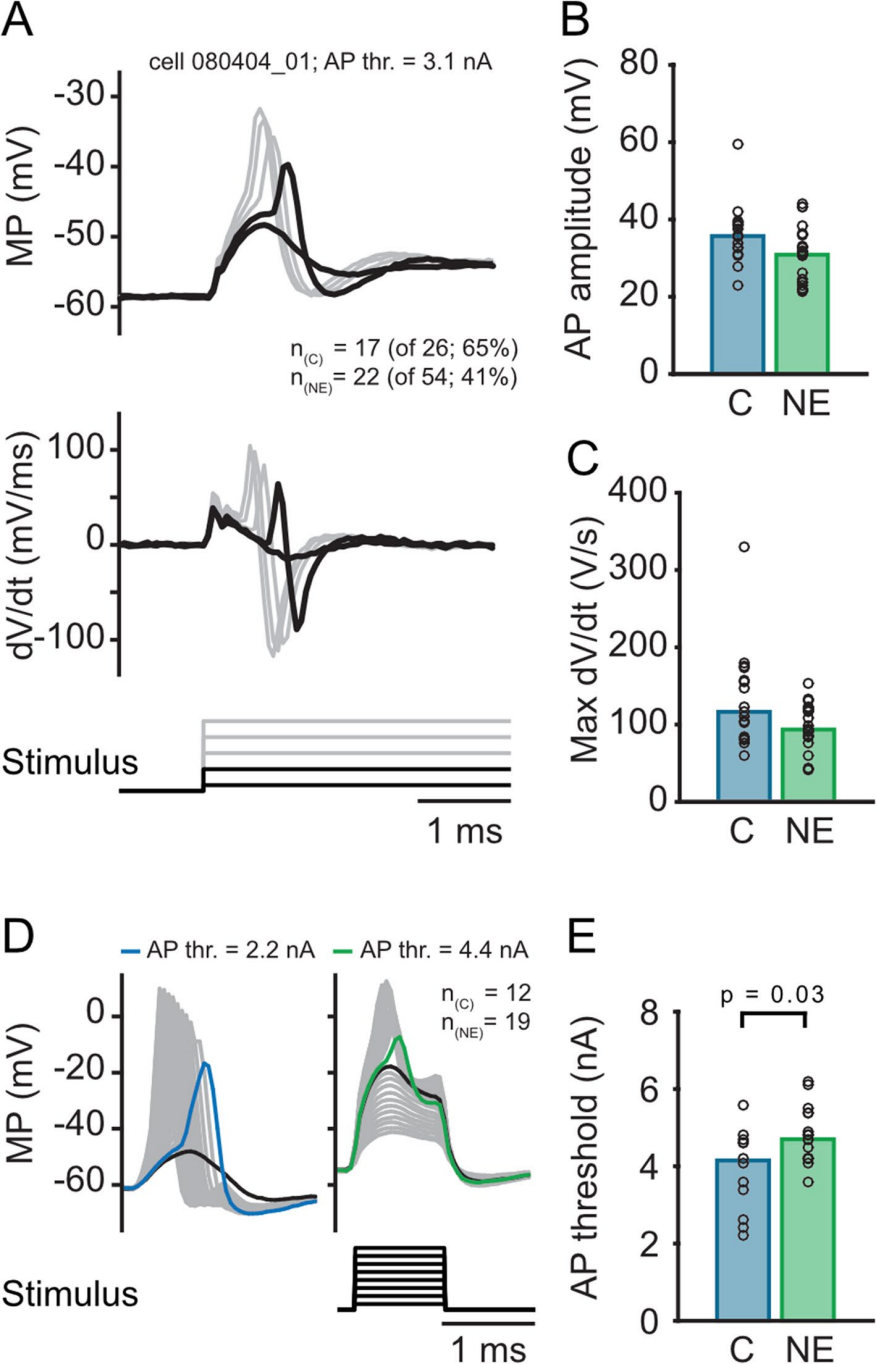
Action potential characteristics and generation in MSO neurons from adult animals with and without noise exposure: Action potentials are getting smaller and faster, and the threshold to generate action potential is higher after noise exposure. (**A**) Example action potentials of a principal MSO neuron induced by high currents (see Fig. 1B). The maximal current of 2.4 nA induced action potential in 65% (n_c_ = 17 of 26 tested) of the neurons of the control group but only in 41% (n_NE_ 22 of 54 tested) neurons of the NE group. The upper graph shows an exemplary recording in mV, the lower graph the first derivative (dV/dt). (**B**) Amplitudes of the action potentials (AP) (c = 35.7 mV; NE = 30.9 mV, p = 0.040, without outlier/grey dot p = 0.069) and slope of the action potentials (**C**) (expressed in dV/dt) (c = 116.5 V/s; NE = 93.6 V/s, p = 0.087, without outlier grey dot p = 0.14) of the two groups. (**D**) Example action potentials evoked by a family of short increasing 1 ms currents (0.1 to 7 nA, 0.1nA steps). This stimulus was used in a subset of MSO neurons to precisely investigate the action potential threshold. (**E**) Neurons that elicited an action potential by a maximal current of 7 nA, showed a significantly higher action potential threshold (c = 4.2 nA; NE = 4.7 nA, p = 0.03) in NE neurons. Bars represent the median values, significance was assessed using two-sided Wilcoxon rank sum test.

Functionally, the lowered R_input_ in MSO neurons might change the action potential threshold recorded in the soma^36^. To probe for changes in action potential threshold, action potentials were initially evoked by a 500 ms long + 2.4 nA current injection (Fig. 2A). With this current intensity action potentials were induced in 65% (17 of 26 tested) of the neurons of the control group but only in 41% (22 of 54 tested) of the NE group. Thus, MSO neurons from NE animals appeared less excitable (Fisher's exact test; p = 0.034). The size of the evoked action potential determined from baseline and the speed of the depolarization (Fig. 2A, bottom, dV/dt) were only slightly smaller but did not significantly differ between control and NE neurons (Fig. 2B, control 35.7, n = 17; NE 30.9, n = 21, p = 0.069, without outlier (n = 22) p = 0.040, two-sided Wilcoxon rank sum test; Fig. 2C, control 116.5 V/s n = 17; NE 93.6 V/s n = 22, p = 0.087, two-sided Wilcoxon rank sum test). To detect differences in action potential current threshold between control and NE neurons in more detail, a short 1 ms current injection was delivered to the soma and increased in 0.1 nA steps up to 7 nA (Fig. 2D). The current injected to drive the first supra-threshold event was significantly larger in NE animals (control 4.2 nA n = 12; NE 4.7 nA n = 19; p = 0.03, two-sided Wilcoxon rank sum test; Fig. 2E). Thus, in accord with a lower somatic R_input_ the current threshold of action potentials increased after noise exposure.

**Figure 3:**
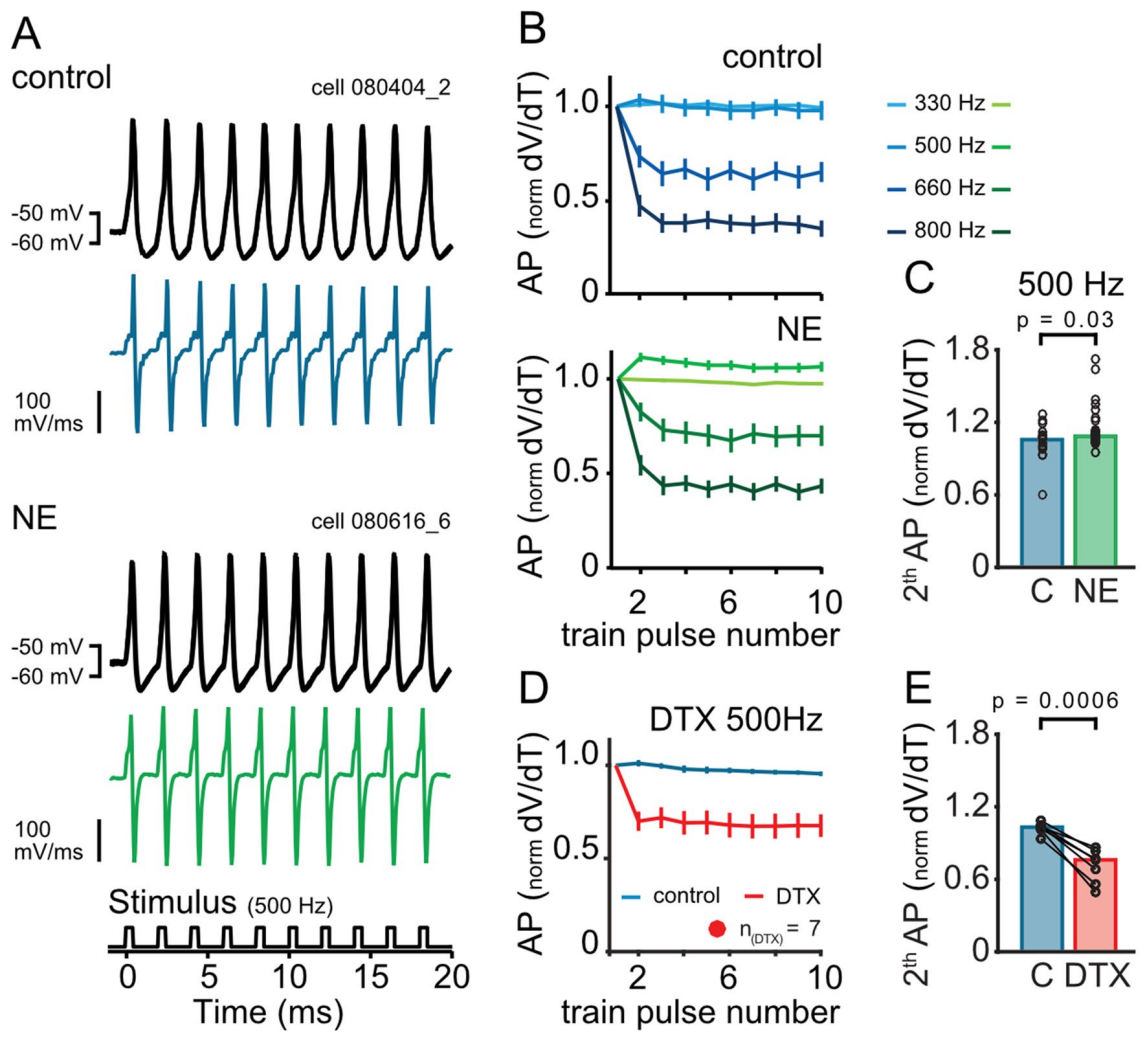
Action potential generation is facilitated at higher frequencies after noise exposure. (**A**) Exemplary raw traces (three repetition) and the first derivative (dV/dt) of the response to a train of 10 stimuli (indicated below). The upper panel shows the data of the control group the lower panel the data of the NE group. (**B**) Mean response of action potential (AP) depolarisation speed (dV/dt) (± SEM normalized to the first action potential of the 10-stimuli train shown for four different frequencies (the control group: upper panel, NE group: bottom panel). (**C**) The rise of the second amplitude is significantly increased at the 500 Hz-train after noise exposure (p = 0.0348). (**D**) Mean action potential depolarisation speed (dV/dt) (± SEM) after blocking the Kv1 channel with DTX (Dendrotoxin 100 nM) for the 10 pulse 500 Hz-stimulus train. (**E**) Action potential depolarisation speed decreases significantly (p = 0.0006) in neurons after DTX application. Bars represent the median values, significance was assessed using two-sided Wilcoxon’s signed rank (E) and two-sided Wilcoxon rank sum test (C).

A change in R_input_ may also alter the likelihood of neurons to generate action potentials during repetitive stimulations. The resting conductance of MSO neurons favors rates of resonance driven output between 300 and 500 Hz^29^. Increased conductance increases the resonance frequency and thus might support higher frequencies of ongoing output generation^29^. To examine whether the increased membrane conductance by noise exposure alters the behavior of repetitive action potential generation, 10 short 0.5 ms long currents were injected 0.5 nA above threshold with frequencies of 330 to 800 Hz. Almost all neurons (control n = 19; NE n = 35) faithfully generated action potentials to each stimulation pulse in the train of up to 500 Hz (Fig. 3A). To investigate the action potential generation the dV/dt values were extracted (Fig. 3A, blue and green traces). Normalizing the dV/dt values to the first event revealed no change of the speed of action potential depolarisation during the 330 Hz train stimulation (Fig. 3B). At higher stimulation frequencies (660 and 800 Hz) a depression of the action potential speed from the second stimulation pulse onwards was observed for both groups. However, at 500 Hz stimulation frequency a small facilitation of the action potential speed was observed, that was significantly larger in NE cells (Fig. 3C, p = 0.03, two-sided Wilcoxon rank sum test). To indicate whether the conductance of the K_LT_ channels could be involved in this facilitation, the same train of current injections was repeated in the presence of 100 nM DTX (n = 7; Fig. 3D) a K_LT_ antagonist in MSO neurons^24,26,28^. Indeed, increasing R_input_ by blocking K_LT_ led to a decrease of the first derivative during 500 Hz stimulations in control animals (Fig. 3E, p = 0,016, two-sided Wilcoxon’s signed rank test). Thus, consistent with the input resistance dependent membrane resonance properties of MSO neurons^29^, the decreased R_input_ after noise experience allows these neurons to follow with higher fidelity high stimulation rates.

### Long-term regulation of spontaneous synaptic transmission

**Figure 4:**
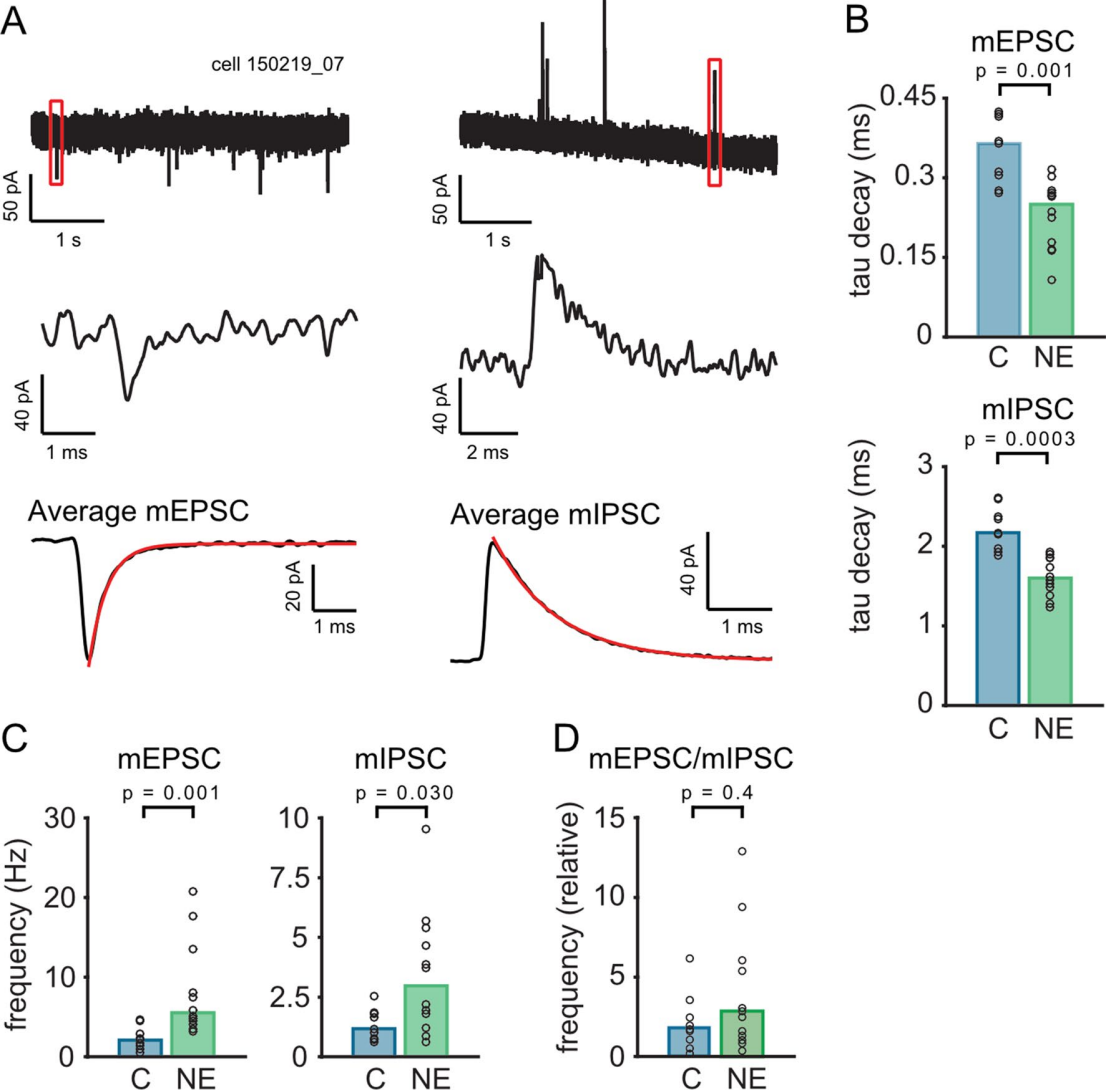
Characterization of miniature EPSCs and IPSCs in MSO neurons from adult animals with and without noise exposure: While the amplitude of the miniature events stays constant, the events are faster and their frequency is increased. (**A**) Recording of mEPSCs (left) and mIPSCs (right) in the same neuron using different holding potentials (− 60 mV for mEPCSs and + 10 mV for mIPSCs). The middle graph shows a single enlarged mEPSC or mIPSC from the recording (upper graph, red box) and the bottom graph the average mEPCS or mIPSC. (**B**) Decay time constants (tau decay) of the average mEPSCs (p = 0.0014) and mIPSCs (p = 0.0003). (**C**) Frequency of mEPSCs (left; p = 0.00095) and mIPSCs (right; p = 0.0302) significantly increases in neurons after noise exposure. (**D**) The relative frequency (left; p = 0.414) of mEPSCs/mIPSCs does not change after noise exposure.

Turning from a comparison of biophysical membrane properties to synaptic inputs we next quantified spontaneous mEPSCs and mIPSCs in control and NE animals. Spontaneous mPSCs were regarded as miniature PSC as TTX does not alter their amplitude^18^. Both mEPSCs and mIPSCs were recorded in the same neurons with different holding potentials (Fig. 4A). Detected events at -70 mV holding potential, corresponding to the reversal of chloride currents were regarded as mEPSCs and events at + 10 mV holding potential, the reversal of glutamatergic current, were regarded as mIPSCs. For each cell, all detected mEPSCs (N_mean_ = 677 ± 225) were averaged. mEPSCs appeared accelerated in NE animals (Fig. 4B), due to a decrease in decay time (control: 0.36 ms; NE: 0.25 m; p = 0.0014, two-sided Wilcoxon rank sum test). Similarly, the mIPSC decay time constant was reduced in NE animals (control: 2.17 ms, n = 9 NE: 1.6 ms, n = 12; p = 0.0003, two-sided Wilcoxon rank sum test; Fig. 4B). Finally, the frequency of mPSCs was determined in the same cells. mEPSCs appeared more frequent in NE animals (control: 2.08 Hz, n = 9; NE: 5.52 Hz, n = 12; p = 0.00095, two-sided Wilcoxon rank sum test; Fig. 4C, left), an increase also observed for mIPSCs (control 1.17 Hz, n = 9; NE 2.97 Hz; n = 12, p = 0.0302, two-sided Wilcoxon rank sum test; Fig. 4C, right). The mutual increase of mEPSC and mIPSC frequency leads to the maintenance of the mEPSC/mIPSC frequency ratio (control 1.78, n = 9; NE 2.72 Hz; n = 12, p = 0.41, two-sided Wilcoxon rank sum test; Fig. 4D). Together, these data indicate that an increase sensory input induced by continuous omnidirectional noise exposure leads to a simultaneous increase of the frequency of the accelerated excitatory and inhibitory mPSC possibly keeping the balance of these inputs constant.

## Discussion

Concurrent noise exposure has a strong impact on the processing of binaural information in the first stages of the auditory pathway^14,32,37–39^. Also, psychophysical studies showed, that sound source localization is modulated by the physical features of the concurrent noise^40–42^. Direct evidence from binaural neurons in the auditory brainstem demonstrated their adaptation of neuronal responses during long-lasting exposure of concurrent noise^14^. Here we describe the physiological cellular adaptations of neurons of the MSO to the same long-lasting, moderate, omnidirectional noise. We show that biophysical changes include a decrease in input resistance, an increase in action potential current threshold, and a change in firing performance to train stimulations. Furthermore, the synaptic inputs to MSO neurons also appear to undergo activity dependent adaptation. Together, we hypothesize that the increased sensitivity may result from homeostatic mechanisms crucial to maintain firing rates in a working range and may guarantee the extraction of spatial information out of the environmental noise.

The noise induced alteration in the sag potential and the decreased in R_input_ indicate an enhanced I_H_ current. Moreover, the decrease in R_input_ together with the increase in action potential current threshold, the change in output performance indicate an enhanced K_LT_ current. The increase in action potential current threshold is consistent with an influence of the DTX sensitive current in action potential generation^34^ corroborating the interpretation that higher current thresholds are mediated by larger DTX-sensitive K_LT_ currents. Both I_H_ and K_LT_ are known to set the input resistance of MSO neurons^35^. As the resting membrane potential is unaffected by the long lasting moderate noise exposure we suggest that both the I_H_ and the K_LT_ currents are enhanced in a balanced way. Such a balanced upregulation might be important to maintain the appropriate interactions of K_LT_ and I_H_ during ongoing activity^43^. The facilitated generation of higher output frequencies after noise induced leak increase agrees with the finding that leakier cells support higher output rates by resonance^29^. An increase in K_LT_ might be crucial towards this adaptation process, as it has been described to enhance the signal-to-noise ratio in MSO neurons^34^. Thus, the decreased R_input_, likely based on K_LT_ and I_H_ appears as a cellular strategy to increase the signal-to-noise ratio to maintain the extraction of relevant binaural cues with the requested accuracy and thereby counterbalancing masking effects of concurrent noise.

A change in mPSC frequency can be attributed to a variety of underlying mechanisms, including a change in release probability, a change in number or the redistribution of synapses. After two weeks in moderate, omnidirectional white noise the frequency of both mIPSC and mEPSC were increased, maintaining the same E/I ratio. The mechanism for the apparently maintained E/I ratio after sound exposure remains elusive. Thus, the balance between excitation and inhibition appears unchanged despite the overall synaptic drive might be enhanced. We can only speculate about the functional impact of the changes in mPSC, as the effect of long-lasting omnidirectional noise exposure on evoked synaptic responses remains elusive. The increased mPSC frequency might indicate also an increased evoked response either in amplitude or width. Such an increase might be crucial to overcome the increased action potential current threshold in the adapted state without jeopardizing the coincidence detection mechanism based on excitation and inhibition^19–22^. The cellular mechanism that leads to this compensatory increase in synaptic drive remains unknown. Generally, moderate noise induced changes of synaptic parameters include the number of release sites and release probability^12^. The molecular targets of the underlying noise induced modification in the auditory brainstem are unclear, but proteinergic changes induced by altered activity levels are manifold in the central auditory pathways^5^. This increase in precision driven by long-lasting noise exposure differs from the short-term mechanisms driven by short adapter sound stimuli that activate the presynaptic GABA_B_ signaling cascade^15^. During these rapidly driven adaptive processes, the time course of mPSCs remained unchanged. Therefore, the alterations in mPSC kinetics are more likely to involve a homeostatic regulation of the postsynaptic mechanism.

Our data and experimental design precludes us from identifying unambiguously the stimulus feature responsible for the observed adaptive changes. One effect of the omnidirectional noise exposure is most likely an increase in neuronal activity. The second effect of this stimulation paradigm is the suppression of the binaural information of low frequency sound sources by masking. Thus, the adaptive changes might be induced by an activity increase or a loss of spatial cue. At least during development both cues appear crucial for the functional maturation of the MSO circuit. The loss of spatial cues hampers the development of inhibition^6,9,44^ while activity likely drives the excitatory development and homeostatic development^45^. Therefore, both cues might be relevant to induced long-term adaptive changes to improve signal detection to counterbalance sound exposure in adult gerbils.

## Material and methods

### Animals and noisebox

All experiments and experimental protocols were in accordance with the relevant guidelines and regulations of German law on the protection of animals and licensed by the local authority the Regierung von Oberbayern after being evaluated, questioned and approved by their ethics committee (Tierschutzgesetz; AZ 2112531-40/01, AZ 55.2-1-54-2531-57-05, and AZ 55.2-1-54.2531-105-10). We investigated two groups of adult (3–4 months old) Mongolian gerbils (*Meriones unguiculatus*) of either sex. All animals were raised in a normal acoustic environment (38–40 dBA). The control group was not exposed to noise (N = 15). A second group, called the NE group was exposed to omnidirectional white noise for 14 days (for details see Siveke, et al.^14^) and tested within the following 7 days (N = 22). Overall, this work complied with the ARRIVE guidelines (https://arriveguidelines.org/).

### Slice preparation, in vitro electrophysiology and data analysis

Brain slices were prepared from gerbils of postnatal day (P) > 60. Animals were anesthetized with isoflurane and decapitated. Brains were removed in dissection solution containing (in mM) 50 sucrose, 25 NaCl, 25 NaHCO_3_, 2.5 KCl, 1.25 NaH_2_PO_4_, 3 MgCl_2_, 0.1 CaCl_2_, 25 glucose, 0.4 ascorbic acid, 3 *myo*-inositol and 2 Na-pyruvate (pH 7.4 when bubbled with 95% O_2_ and 5% CO_2_). Subsequent to brain removal, 90–110 µm horizontal slices were taken with a VT1200S vibratome (Leica). Slices were incubated in recording solution (same as slice solution but with 125 mM NaCl, no sucrose and 2 mM CaCl_2_ and 1 mM MgCl_2_ at 36 °C for 15–45 min, bubbled with 5% CO_2_ and 95% O_2_). After incubation, slices were transferred to a recording chamber attached to a microscope (BX50WI; Olympus) equipped with gradient contrast illumination and continuously perfused with recording solution. Cells were visualized with a TILL Photonics system composed of a VGA CCD camera, a monochromator and its control unit. Whole-cell recordings were performed using an EPC10/2 amplifier (HEKA Elektronik) at 34–36 °C. Data were acquired at 50 kHz and for mPSCs at 100 kHz and always low-pass filtered at 3 kHz. For voltage clamp recordings, the access resistances were compensated to a residual of 3 MOhm. For current clamp recordings, the bridge balance was set to 100% after estimating the access resistance in voltage clamp. Clamp conditions were monitored throughout the experiment. For current clamp recordings, the internal solution contained (in mM): 145 K-gluconate, 5 KCl, 2 Mg-ATP, 2 K-ATP, 0.3 Mg-GTP, 10 Na-phosphocreatine, 10 HEPES, 5 EGTA (osmolarity of 300 and ph of 7.25 adjusted with KOH). For voltage clamp recordings, the internal solution contained (in mM): 135 Cs-gluconate, 10 TEACl, 2 Mg-ATP, 2 K-ATP, 0.3 Na_2_-GTP, 10 Na_2_-phosphocreatine, 10 HEPES, 5 Cs-EGTA, 0.01 ZD7288, 5 Qx-314-Br (osmolarity of 300 and ph of 7.1 adjusted with CsOH). No liquid junction potential correction was made. Miniature excitatory and inhibitory post-synaptic currents (mEPSCs and mIPSCs) were recorded either at the reversal potential of the excitatory (+ 10 mV) or inhibitory current (− 70 mV) respectively. To visualize the recorded cells during and after the recordings, all internal solutions included 50 µM Alexa 594 (molecular probes, USA). After fixation of the slices in 4% paraformaldehyde solution for 10–20 h the filled neuron’s location and bipolar shape^46^ was histologically verified using standard fluorescent microscopy. Data was analyzed offline using custom written functions in IgorPro (Wavemetrics, OR, USA). Peak and steady-state I-V plots were generated from voltage response to 1000 ms current steps (− 2.5 to 4.3 nA, 0.4 pA steps, 3 repetitions) measured at the beginning (8–13 ms) and during the last 40 ms of the step pulse, respectively (see Fig. 1B). The membrane resting potential was quantified at the beginning of the first 5 ms of the recordings and averaged over all 18 current steps and the 3 repetitions. The input resistance (R_input_) was obtained from the slope of the mean I–V plot between 5 and 20 mV below rest (see Fig. 1C). In addition, for each cell separately the R_input_ (steady-state) was obtained from the average voltage response elicited by a 50-ms hyperpolarizing step pulse (− 0.1 nA, 30 repetitions, see Fig. 1D). The fastest slope of the generated action potential was measured as the peak of the differentiated voltage trace (dV/dt, see Fig. 2A). Both, peak amplitude and the dV/dt value of the action potential were obtained from the 1000 ms current step (4.3 nA, see Fig. 2A–C). The cell specific action potential current thresholds were more accurately determinate using a short 1-ms depolarizing steps rising from 2 to 7 nA (0.2 nA steps, 3 repetitions, see Fig. 2D). Spontaneous, miniature mPSCs were extracted based on the template-matching algorithm of Clements and Bekkers^47^ implemented by Taschenberger^48^ in an IGOR routine. The template for mEPSC was 4 ms long and consisted of a 100 µs rise time and a 100 µs fast and 100 µs fast and slow decay time constant generating a mono-exponential decay. For mIPSC matching the template was 10 ms long and consisted of a 100 µs rise time and a 1 ms fast and 1 ms slow decay time constant. The detection threshold was adjusted to the noise level of each recording and thus allowed the extraction of false positive to ensure that all detectable mPSCs were captured. Post-hoc mPSC sorting to remove false positive events and mPSC analysis was facilitated by implemented Igor routines^18^. Statistical significance was assessed by two-sided Wilcoxon signed rank test for paired samples and the two-sided Wilcoxon rank sum test for independent samples and by the Fisher`s exact test of the null hypothesis (Matlab).

## Data Availability

The data that support the findings to this study are available from the corresponding authors upon request.

## Abbreviations

mEPSC: Miniature excitatory post-synaptic current
mIPSC: Miniature inhibitory post-synaptic current
MSO: Medial superior olive
DTX: Dendrotoxin
